# Supplementary material for: Models of Risk Selection in Maternal and Newborn Care: Exploring the Organization of Tasks and Responsibilities of Primary Care Midwives and Obstetricians in Risk Selection across The Netherlands
Source: Int J Environ Res Public Health. 2022 Jan 18;19(3):1046. doi: 10.3390/ijerph19031046 (PMC8834427; doi:10.3390/ijerph19031046)
Supplement: Supplementary file 1 [file ijerph-19-01046-s001.zip › ijerph-1474084-supplementary.pdf]

Supplementary Materials

Table S1. Moment of routinely scheduled discussions, attending professionals, and the way in which routinely scheduled discussions take place (*n*,%).

	**Bi-Disciplinary**	**Multidisciplinary**
	n (%)	*n* (%)
Total	58 (100)	58 (100)
Moment of discussions		
Moment in the care pathway		
After the booking appointment	19 (32.76)	1 (2.70)
15 weeks gestation	1 (1.75)	
16 weeks gestation	1 (1.75)	
20 weeks gestation		18 (48.65)
30 weeks gestation	1 (1.75)	
34 weeks gestation		1 (2.70)
Third trimester		1 (2.70)
Frequency		
Weekly	6 (10.53)	1 (2.70)
Every two weeks	8 (14.04)	
Every three weeks	1 (1.75)	
Every four weeks	1 (1.75)	
Every six weeks	6 (10.53)	
Unclear	1 (1.75)	2 (5.41)
Contradictory answers amongst respondents within the MCC	1 (1.75)	13 (35.14)
Attending professionals		
Primary care midwife	56 (96.55)	35 (94.59)
Hospital-based midwife	25 (43.10)	27 (72.97)
Obstetrician	56 (96.55)	34 (91.89)
Resident obstetrician	13 (22.41)	17 (45.95)
Paediatrician		33 (89.20)
Maternity care assistant		9 (24.32)
General practitioner		3 (8.11)
Obstetric nurse		8 (21.62)
Social worker		29 (78.38)
Child healthcare nurse		18 (48.65)
Other professional		12 (32.43)
Unclear	2 (3.45)	2 (5.41)
The way in which discussions take place		
Face to face at the midwifery practice	12 (20.69)	1 (2.70)
Face to face at the hospital	47 (81.03)	33 (89.20)
By phone	27 (46.55)	6 (16.22)
By regular e-mail	1 (1.72)	
By encrypted e-mail	5 (8.62)	2 (5.41)
By video call	4 (6.90)	

Other way	2 (3.45)	2 (5.41)
Unclear	1 (1.72)	2 (5.41)

Table S2. Reasons of nine maternal care collaborations for reversing the change in the usual organization of tasks and responsibilities in risk selection.

MCCs * (*n*)	Change in the usual organization of tasks and responsibilities in risk selection, and reason for reversing the change.
1	*Organization of the booking appointment:* all women with uncomplicated pregnancies start their care in primary midwifery care practices, and women with a high-risk profile start their care in secondary obstetrician-led care in the hospital. Primary care midwives are responsible for the booking appointment of women with uncomplicated pregnancies, and obstetricians are responsible for the booking appointment of women with a high-risk profile.<br>*Changed to*: all booking appointments by primary care midwives.<br>*Changed back*: usual model of care.<br>*Reason*: logistical reasons.
4	*Organization of risk assessment after the booking appointment:* primary care midwives assess risk and initiate a consultation or transfer of care to obstetrician-led care in the hospital only when necessary, without discussing this first with the obstetrician.<br>*Changed to*: primary care midwives and obstetricians discuss all women who had a booking appointment with a primary care midwife whether a consultation or transfer of care is to obstetrician-led care in the hospital is indicated. Moment of discussion is scheduled routinely.
2	*Changed back*: some primary midwifery care practices.<br>*Reason:* time constraints, loss of autonomy, and reasons unclear.
2	*Changed back*: all primary midwifery care practices.<br>*Reason:* time constraints, and change had no added value.
1	*Organization of the booking appointment, risk assessment after booking appointment, and moment of discussion:* all women with uncomplicated pregnancies start their care in primary midwifery care practices, and women with a high-risk profile start their care in secondary obstetrician-led care in the hospital. Primary care midwives are responsible for the booking appointment of women with uncomplicated pregnancies, and obstetricians are responsible for the booking appointment of women with a high-risk profile. Primary care midwives assess risk and initiate a consultation or transfer of care only when necessary, without discussing this first with the obstetrician. Moment of discussion between primary care midwives and obstetricians is only scheduled at request, and not scheduled routinely.<br>*Changed to*: all booking appointments by primary care midwives. Primary care midwives and obstetricians discuss all women who had a booking appointment with a primary care midwife whether a consultation or transfer of care is to obstetrician-led care in the hospital is indicated. Moment of discussion is scheduled routinely.<br>*Stopped*: primary care midwives and obstetricians discuss all women who had a booking appointment with a primary care midwife whether a consultation or transfer of care to obstetrician-led care in the hospital is indicated. Moment of discussion is scheduled routinely.<br>*Preserved*: all booking appointments by primary care midwives.<br>*Reason*: time constraints, financial constraints, and change had no added value.
2	*Organization of risk assessment after booking appointment, and moment of discussion:* primary care midwives assess risk and initiate a consultation or transfer of care only when necessary, without discussing this first with the obstetrician. The moment of discussion between primary care midwives and obstetricians is only scheduled at request, and not scheduled routinely.
1	*Changed to*: primary care midwives and obstetricians discuss all women who had a booking appointment with a primary care midwife whether a consultation or transfer of care to obstetrician-led care in the hospital is indicated. Moment of discussion is scheduled routinely.<br>*Stopped*: primary care midwives and obstetricians discuss all women who had a booking appointment with a primary care midwife whether a consultation or transfer of care is to obstetrician-led care in the hospital is indicated.<br>*Preserved*: routinely scheduled moment of discussion between primary care midwife and obstetrician.<br>*Reason*: time constraints and finances constraints.
1	*Changed to*: primary care midwives and obstetricians discuss whether a consultation or transfer of care is indicated for all women who had a booking appointment with a primary care midwife. Women with psychosocial complications are discussed in a multidisciplinary way. Both discussions are scheduled routinely.<br>*Stopped*: primary care midwives and obstetricians discuss all women who had a booking appointment with a primary care midwife whether a consultation or transfer of care is to obstetrician-led care in the hospital is indicated. Moment of discussion is scheduled routinely.<br>*Preserved*: women with psychosocial complications are discussed multidisciplinary at a routinely scheduled moment.

	Reason: change had no added value.
1	*Organization of the booking appointment, and moment of discussion:* all women with uncomplicated pregnancies start their care in primary midwifery care practices, and women with an high risk profile start their care in secondary obstetrician-led care in the hospital. Primary care midwives are responsible for the booking appointment for women with uncomplicated pregnancies and obstetricians are responsible for the booking appointment of women with a high-risk profile. Moment of discussion between primary care midwives and obstetricians is only scheduled at request, and not scheduled routinely.<br>*Changed to*: all booking appointments by primary care midwives. Primary care midwives and obstetricians discuss all women who had a booking appointment with a primary care midwife a consultation or transfer of care is to obstetrician-led care in the hospital is indicated. Moment of discussion is scheduled routinely.<br>*Stopped*: primary care midwives and obstetricians discuss all women who had a booking appointment with a primary care midwife whether a consultation or transfer of care is to obstetrician-led care in the hospital is indicated. Moment of discussion is planned routinely.<br>*Preserved*: all booking appointments by primary care midwives.<br>*Reason*: time constraints and finances constraints.

* MCC = Maternity Care Collaboration.

Table S3. Levels of satisfaction related to the model of care (*n*,%).

	**Total**			**MRS^ 1**			**MRS 2**			**MRS 3**		
	MCC +	P *	O #	MCC	P	O	MCC	P	O	MCC	P	O
	n (%)	n (%)	n (%)	n (%)	n (%)	n (%)	n (%)	n (%)	n (%)	n (%)	n (%)	n (%)
Total	69 (100)	67 (100)	53 (100)	42 (100)	41 (100)	30 (100)	16 (100)	15 (100)	14 (100)	11 (100)	11 (100)	8 (100)
Quality of care												
Very satisfied	7 (10)	6 (9)	12 (23)	3 (7)	4 (10)	6 (20)	3 (19)	1 (7)	5 (36)	1 (9)	1 (9)	1 (13)
Satisfied	49 (71)	50 (75)	24 (46)	29 (69)	30 (73)	11 (37)	11 (69)	12 (80)	8 (57)	9 (82)	8 (72)	5 (63)
A little satisfied	12 (17)	10 (15)	12 (23)	9 (21)	6 (15)	9 (30)	2 (13)	2 (13)	1 (7)	1 (8)	2 (18)	2 (25)
Neutral	1 (1)	1 (1)	1 (2)	1 (2)	1 (2)	1 (3)	0	0	0	0	0	0
A little unsatisfied	0	0	2 (4)	0	0	1 (3)	0	0	0	0	0	0
Unsatisfied	0	0	1 (2)	0	0	2 (7)	0	0	0	0	0	0
Very unsatisfied	0	0	0	0	0	0	0	0	0	0	0	0
Quality of collaboration												
Very satisfied	6 (9)	6 (9)	7 (13)	3 (7)	3 (7)	4 (13)	2 (13)	2 (13)	2 (14)	1 (9)	1 (9)	1 (13)
Satisfied	45 (65)	42 (63)	31 (59)	25 (60)	23 (56)	16 (53)	11 (69)	11 (73)	10 (71)	9 (82)	8 (73)	5 (63)
A little satisfied	17 (25)	19 (28)	12 (23)	13 (31)	15 (37)	8 (27)	3 (19)	2 (13)	2 (14)	1 (9)	2 (18)	2 (25)
Neutral	1 (1)	0	2 (4)	1 (2)	0	0	0	0	0	0	0	0
A little unsatisfied	0	0	0	0	0	2 (7)	0	0	0	0	0	0
Unsatisfied	0	0	0	0	0	0	0	0	0	0	0	0
Very unsatisfied	0	0	0	0	0	0	0	0	0	0	0	0
Organization of care												
Very satisfied	2 (3)	1 (1)	7 (13)	1 (2)	0	3 (10)	1 (6)	1 (7)	2 (15)	0	0	2 (25)
Satisfied	34 (49)	34 (51)	21 (40)	19 (45)	22 (54)	12 (30)	8 (50)	6 (40)	6 (43)	7 (64)	6 (55)	3 (38)
A little satisfied	32 (46)	31 (46)	19 (37)	21 (40)	18 (44)	10 (33)	7 (44)	8 (53)	6 (43)	4 (36)	5 (45)	3 (38)
Neutral	1 (1)	0	3 (6)	1 (2)	0	3 (10)	0	0	0	0	0	0
A little unsatisfied	0	1 (1)	1 (2)	0	1 (2)	1 (3)	0	0	0	0	0	0
Unsatisfied	0	0	1 (2)	0	0	1 (3)	0	0	0	0	0	0
Very unsatisfied	0	0	0	0	0	0	0	0	0	0	0	0
Time investment												
Very satisfied	1 (1)	1 (1)	4 (8)	1 (2)	1 (2)	2 (7)	0	0	1 (7)	0	0	1 (13)
Satisfied	17 (25)	13 (19)	18 (45)	11 (16)	8 (20)	12 (40)	3 (19)	3 (20)	4 (29)	3 (27)	2 (18)	2 (25)
A little satisfied	38 (55)	37 (55)	15 (29)	22 (52)	23 (56)	5 (17)	9 (56)	6 (4)	7 (15)	7 (64)	8 (73)	3 (38)

Neutral	9 (13)	13 (19)	1 (13)	6 (14)	7 (17)	4 (13)	2 (13)	5 (33)	0	0	1 (9)	1 (13)
A little unsatisfied	3 (4)	1 (1)	0	1 (2)	1 (2)	2 (7)	2 (13)	0	1 (7)	1 (9)	0	0
Unsatisfied	1 (1)	2 (3)	1 (13)	1 (2)	1 (2)	5 (17)	0	1 (7)	1 (7)	0	0	1 (13)
Very unsatisfied	0	0	0	0	0	0	0	0	0	0	0	0
Autonomy												
Very satisfied	5 (7)	4 (6)	14 (27)	4 (10)	3 (7)	8 (27)	1 (6)	1 (7)	4 (29)	0	0	2 (25)
Satisfied	45 (65)	45 (67)	28 (54)	28 (67)	29 (71)	15 (50)	10 (63)	10 (67)	7 (50)	7 (64)	6 (55)	6 (75)
A little satisfied	19 (28)	16 (24)	8 (15)	10 (24)	9 (22)	5 (17)	5 (31)	3 (20)	3 (21)	4 (36)	4 (47)	0
Neutral	0	1 (1)	2 (4)	0	0	2 (7)	0	0	0	0	1 (9)	0
A little unsatisfied	0	1 (1)	0	0	0	0	0	1 (7)	0	0	0	0
Unsatisfied	0	0	0	0	0	0	0	0	0	0	0	0
Very unsatisfied	0	0	0	0	0	0	0	0	0	0	0	0

[+] MCC = Maternity Care Collaboration. ^ MRS = Model of risk selection. [*]P = Primary midwifery care practices. [#]O = Obstetrics departments.
